# Supplementary material for: Utility of Comprehensive Serum Glycopeptide Spectra Analysis (CSGSA) for the Detection of Early Stage Epithelial Ovarian Cancer
Source: Cancers (Basel). 2020 Aug 21;12(9):2374. doi: 10.3390/cancers12092374 (PMC7563232; doi:10.3390/cancers12092374)
Supplement: Supplementary file 1 [file cancers-12-02374-s001.zip › cancers-874799-supplementary.docx]

Supplementary Materials: Utility of Comprehensive Serum Glycopeptide Spectra Analysis (CSGSA) for the Detection of Early Stage Epithelial Ovarian Cancer

Koji Matsuo, Kazuhiro Tanabe, Masaru Hayashi, Masae Ikeda, Miwa Yasaka, Hiroko Machida, Masako Shida, Kenji Sato, Hiroshi Yoshida, Takeshi Hirasawa, Tadashi Imanishi and Mikio Mikami

| 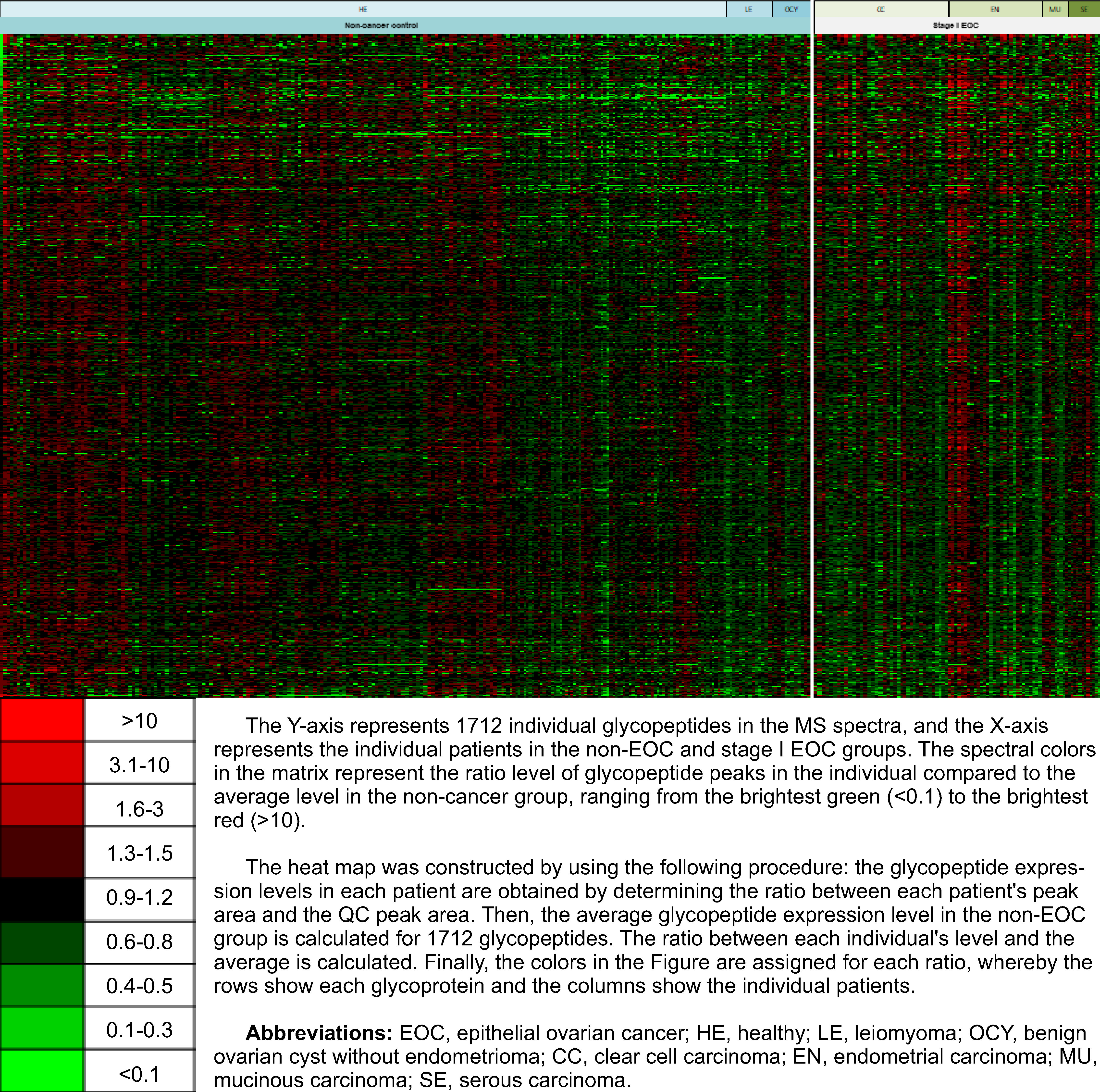 |
| --- |
|  |
| 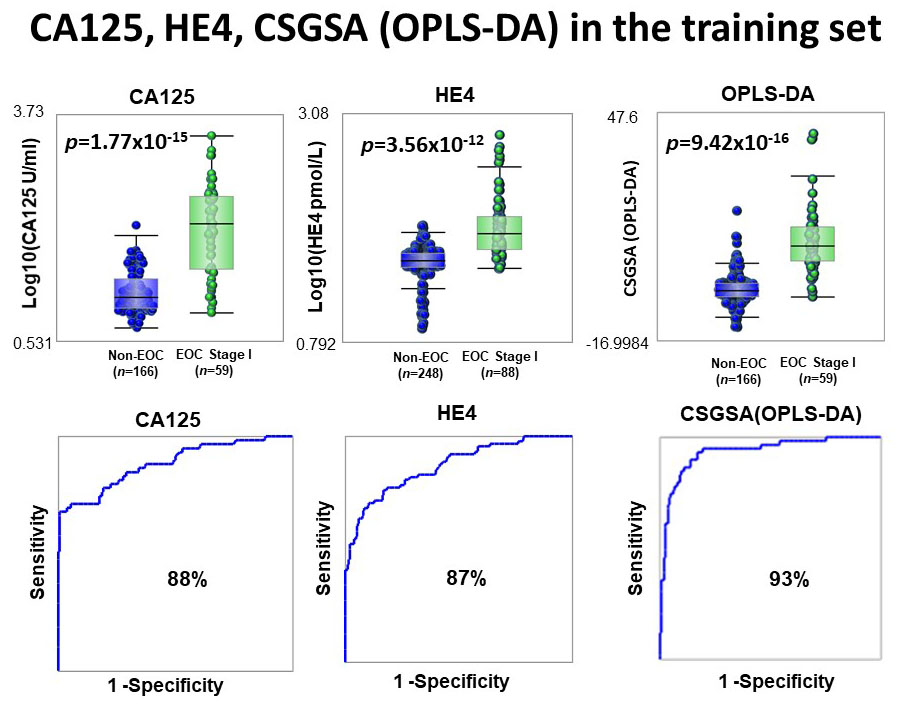 |
|  |

**Figure S1.** Glycopeptide heat map (stage I EOC vs. non-EOC control). The *Y*-axis represents 1712 individual glycopeptides in the MS spectra, and the *X*-axis represents the individual patients in the non-EOC and stage I EOC groups. The spectral colors in the matrix represent the ratio level of glycopeptide peaks in the individual compared to the average level in the non-cancer group, ranging from the brightest green (<0.1) to the brightest red (>10). The heat map was constructed by using the following procedure: the glycopeptide expression levels in each patient are obtained by determining the ratio between each patient’s peak area and the QC peak area. Then, the average glycopeptide expression level in the non-EOC group is calculated for 1712 glycopeptides. The ratio between each individual’s level and the average is calculated. Finally, the colors in the Figure are assigned for each ratio, whereby the rows show each glycoprotein and the columns show the individual patients. Abbreviations: EOC, epithelial ovarian cancer; HE, healthy; LE, leiomyoma; OCY, benign ovarian cyst without endometrioma; CC, clear cell carcinoma; EN, endometrial carcinoma; MU, mucinous carcinoma; SE, serous carcinoma.

**
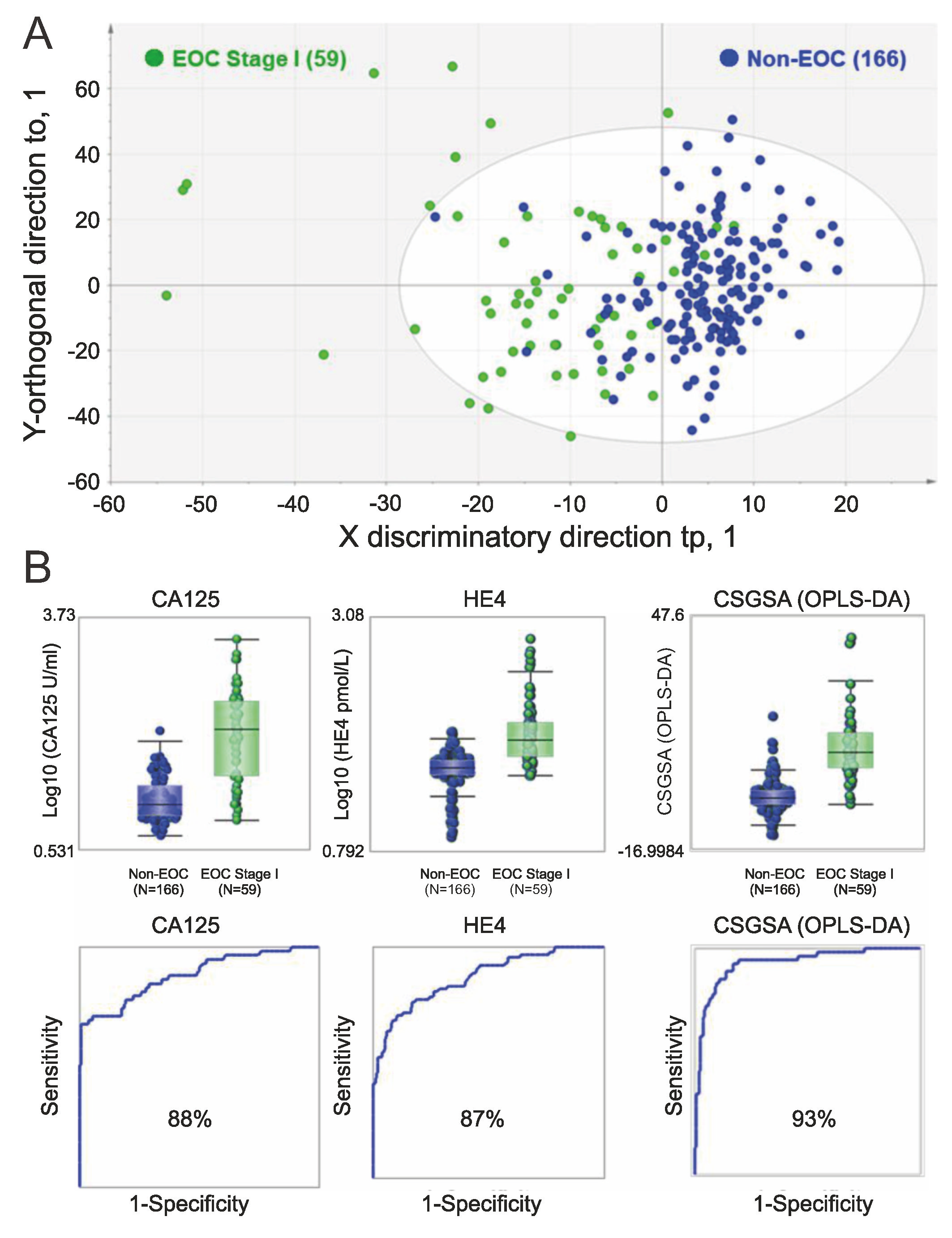
**

**Figure S2.** Training set: Biomarker performance for the detection of stage I EOC vs. non-EOC control. Panel (**A**) shows the glycopeptide spectral data of the stage I EOC group (*n* = 59) and the non-EOC group (*n* = 166) from the training set. OPLS-DA scatter plot was obtained using stage I EOC (green dots) and non-EOC (blue dots) samples. Clearly, a better separation of the patients with stage I EOC and the non-cancer patients was achieved via the supervised orthogonal partial least-squares discriminant analysis (OPLS-DA) model. Internal validation was performed to assess the predictive ability of the corresponding OPLS-DA model with the calculated output (R2 = 47.4%, Q2 (cum) = 43.0%), suggesting a good fit of the model. The R2 value is defined as the proportion of variance in the data obtained by the models and the overall goodness of fit, and the Q2 value is defined as the proportion of variance in the data predictable by the model and the overall cross validation coefficient. CSGSA values indicate the distance from point zero, which is the mean value of all glycopeptide values. Panel (**B**) shows the box-Whisker plot and ROC curve analysis of CA125, HE4, and CSGSA (OPLS-DA) of serum samples obtained from patients with stage I EOC (green dots) and non-EOC patients (blue dots). *p* < 0.001 was used to differentiate between stage I EOC and non-cancer controls for CA-125, HE4, and CSGSA (OPLS-DA). CSGSA values were obtained by using the first OPLS-DA score component, which showed maximum separation of the two groups. CSGSA values indicated the distance from point zero (**A**), which is the mean value of all the glycopeptide values. A summary of the statistical outputs is shown in Table 1.

Definition of the CSGSA (OPLS-DA) Value

OPLS-DA utilizes class information to maximize the separation between classes and to minimize the separation within the groups. The scores plot of OPLS-DA demonstrated that all samples were clearly classified into two groups: EOC and non-EOC. The data structure is constructed by using 1712 glycopeptide levels obtained from 225 examinees (training set; 1712 × 225 matrix), which is geometrically assumed to be 225 scattered plots in 1712-dimension space. OPLS-DA is used to find new two axes in the 1712-dimension space, which maximizes the separation of the two groups (EOC and non-EOC). CSGSA values were obtained by using the first OPLS-DA score component, which showed maximum separation of the two groups.

**Table S1.** Patient characteristics.

| **Characteristic** | **Total** | **Training Set** | **Test Set** | ***p-*Value** |
| --- | --- | --- | --- | --- |
| Number | *n* = 336 | *n* = 225 | *n* = 111 |  |
| EOC group | *n* = 88 | *n* = 59 | *n* = 29 |  |
| Age |  | 54.6 (12.3) | 51.9 (13.2) | 0.916 |
| Histopathology |  |  |  | 0.417 |
| Serous | 13 | 10 | 3 |  |
| Clear cell | 38 | 22 | 16 |  |
| Endometrioid | 28 | 21 | 7 |  |
| Mucinous | 9 | 6 | 3 |  |
| Control group | *n* = 248 | *n* = 166 | *n* = 82 |  |
| Age |  | 54.4 (11.1) | 51.8 (11.5) | 0.881 |
| Healthy control | 220 | 147 | 73 | 0.917 |
| Leiomyoma | 14 | 9 | 5 |  |
| Benign ovarian cyst | 14 | 10 | 4 |  |

Data are given as number or mean age (standard deviation). Abbreviation: EOC, epithelial ovarian cancer.

**Table S2.** Frequency tables based on cutoff values (stage I EOC *vs.* non-EOC control).

| **Training Set (*n* = 225)** | | | | | | | **Test Set (*n* = 111)** | | | | | | |
| --- | --- | --- | --- | --- | --- | --- | --- | --- | --- | --- | --- | --- | --- |
| **CA125 (Training) (Cutoff = 28)** | | | | | | | **CA125 (Test) (Cutoff = 28)** | | | | | | |
|  | | **Condition** | | **Total** |  | |  | | **Condition** | | **Total** |  | |
|  |  | **EOC**  **Stage 1** | **Non-EOC** |  |  |  |  |  | **EOC**  **Stage 1** | **Non-EOC** |  |  |  |
| OPLS-DA | Pos | 46 | 37 | 83 | PPV | 55% | OPLS-DA | Pos | 23 | 16 | 39 | PPV | 59% |
|  | Neg | 13 | 129 | 142 | NPV | 91% |  | Neg | 6 | 66 | 72 | NPV | 92% |
| Total | | 59 | 166 | 225 |  |  | Total | | 29 | 82 | 111 |  |  |
|  | | Sens | Spec |  | Accuracy | |  | | Sens | Spec |  | Accuracy | |
|  |  | 78% | 78% |  | **78%** | |  |  | **79%** | 80% |  | **80%** | |
| **HE4 (Training) (Cutoff = 53)** | | | | | | | **HE4 (Test) (Cutoff = 53)** | | | | | | |
|  | | **Condition** | | **Total** |  | |  | | **Condition** | | **Total** |  | |
|  |  | **EOC**  **Stage 1** | **Non-EOC** |  |  |  |  |  | **EOC**  **Stage 1** | **Non-EOC** |  |  |  |
| OPLS-DA | Pos | 46 | 37 | 83 | PPV | 55% | OPLS-DA | Pos | 23 | 12 | 35 | PPV | 66% |
|  | Neg | 13 | 129 | 142 | NPV | 91% |  | Neg | 6 | 70 | 76 | NPV | 92% |
| Total | | 59 | 166 | 225 |  |  | Total | | 29 | 82 | 111 |  |  |
|  | | Sens | Spec |  | Accuracy | |  | | Sens | Spec |  | Accuracy | |
|  |  | 73% | 82% |  | **80%** | |  |  | 79% | 85% |  | **84%** | |
| **OPLS-DA (Training) (Cutoff = 0.8)** | | | | | | | **OPLS-DA (Test) (Cutoff = 0.8)** | | | | | | |
|  | | **Condition** | | **Total** |  | |  | | **Condition** | | **Total** |  | |
|  |  | **EOC**  **Stage 1** | **Non-EOC** |  |  |  |  |  | **EOC**  **Stage 1** | **Non-EOC** |  |  |  |
| OPLS-DA | Pos | 43 | 30 | 73 | PPV | 59% | OPLS-DA | Pos | 25 | 13 | 38 | PPV | 66% |
|  | Neg | 16 | 136 | 152 | NPV | 89% |  | Neg | 4 | 69 | 73 | NPV | 95% |
| Total | | 59 | 166 | 225 |  |  | Total | | 29 | 82 | 111 |  |  |
|  | | Sens | Spec |  | Accuracy | |  | | Sens | Spec |  | Accuracy | |
|  |  | 90% | 86% |  | **87%** | |  |  | **86%** | 84% |  | **85%** | |
| **Combination (Training) (Cutoff = 0.12)** | | | | | | | **Combination (Test) (Cutoff = 0.12)** | | | | | | |
|  | | **Condition** | | **Total** |  | |  | | **Condition** | | **Total** |  | |
|  |  | **EOC**  **Stage 1** | **Non-EOC** |  |  |  |  |  | **EOC**  **Stage 1** | **Non-EOC** |  |  |  |
| OPLS-DA | Pos | 54 | 12 | 66 | PPV | 82% | OPLS-DA | Pos | 26 | 6 | 32 | PPV | 81% |
|  | Neg | 5 | 154 | 159 | NPV | 97% |  | Neg | 3 | 76 | 79 | NPV | 96% |
| Total | | 59 | 166 | 225 |  |  | Total | | 29 | 82 | 111 |  |  |
|  | | Sens | Spec |  | Accuracy | |  | | Sens | Spec |  | Accuracy | |
|  |  | 92% | 93% |  | **92%** | |  |  | 90% | 93% |  | **92%** | |

The following cutoffs were determined per the ROC curves to discriminate between stage I EOC and non-EOC control in the training set: 28 for CA125, 53 for HE4, 0.8 for CSGSA (OPLS-DA), and 0.12 for the combination model. The summary of the diagnostic performance is shown in Table 1. * The combination index was calculated using CSGSA (OPLS-DA) with CA125 and HE4, as follows: Combination index = (0.43 × CA125) + (0.11 × HE4) + (0.46 × CSGSA [OPLS-DA]). Abbreviations: EOC, epithelial ovarian cancer; Sens, sensitivity; Spec, specificity; PPV, positive predictive value; NPV, negative predictive value; Pos, positive (above cutoff); Neg, negative (below cutoff); CA125, cancer antigen 125; HE4, human epididymis protein 4; CSGSA, comprehensive serum glycopeptide spectra analysis; OPLS-DA, orthogonal partial least-squares discriminant modeling.

**Table S3.** Exclusion criteria for the study.

| **Item** | **Description** |
| --- | --- |
| 1 | A history of hormonal agent administration owing to a malignant tumor, autoimmune disease, and thyroid abnormality |
| 2 | Concurrent malignancy |
| 3 | Abnormal laboratory test result: white blood cell count > 9600 cells/μL; platelet count <48 × 10^3^/μL; lactate dehydrogenase level >263 IU/L; hemoglobin level <9.2 g/dL; and C-reactive protein level >3.0 mg/dL |
| 4 | Renal dysfunction |
| 5 | Liver dysfunction |
| 6 | Age ≥75 years |
| 7 | Mixed histology types |
| 8 | Fallopian tube cancer or peritoneal cancer |
| 9 | Massive ascites or pleural effusion |
| 10 | Diagnosis of endometrioma, mucinous cystadenoma, ovarian germ cell tumor, or sex cord-stromal tumor |

| 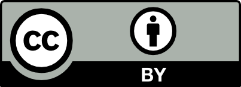 | © 2020 by the authors. Licensee MDPI, Basel, Switzerland. This article is an open access article distributed under the terms and conditions of the Creative Commons Attribution (CC BY) license (http://creativecommons.org/licenses/by/4.0/). |
| --- | --- |
